# An improved high-quality draft genome sequence of *Carnobacterium inhibens* subsp. *inhibens* strain K1^T^

**DOI:** 10.1186/s40793-016-0193-3

**Published:** 2016-09-08

**Authors:** Wayne L. Nicholson, Christina L. Davis, Nicole Shapiro, Marcel Huntemann, Alicia Clum, T. B. K. Reddy, Manoj Pillay, Victor Markowitz, Neha Varghese, Amrita Pati, Natalia Ivanova, Nikos Kyrpides, Tanja Woyke

**Affiliations:** 1 Department of Microbiology and Cell Science, Institute of Food and Agricultural Sciences, University of Florida, Merritt Island, FL 32953 USA; 2 DOE Joint Genome Institute, Walnut Creek, CA USA; 3 Biological Data Management and Technology Center, Lawrence Berkeley National Laboratory, Berkeley, CA USA; 4 Department of Biological Sciences, Faculty of Science, King Abdulaziz University, Jeddah, Saudi Arabia

**Keywords:** *Carnobacterium inhibens* subsp. *inhibens* strain K1^T^

## Abstract

Despite their ubiquity and their involvement in food spoilage, the genus *Carnobacterium* remains rather sparsely characterized at the genome level. *Carnobacterium inhibens* K1^T^ is a member of the *Carnobacteriaceae* family within the class *Bacilli*. This strain is a Gram-positive, rod-shaped bacterium isolated from the intestine of an Atlantic salmon. The present study determined the genome sequence and annotation of *Carnobacterium inhibens* K1^T^. The genome comprised 2,748,608 bp with a G + C content of 34.85 %, which included 2621 protein-coding genes and 116 RNA genes. The strain contained five contigs corresponding to presumptive plasmids of sizes: 19,036; 24,250; 26,581; 65,272; and 65,904 bp.

## Introduction

The genus *Carnobacterium* was proposed in 1987 to encompass a group of closely related bacteria originally classified as unusual species of *Lactobacillus* [1, 2]. The genus *Carnobacterium* includes heterofermentative, facultatively anaerobic, psychrotolerant, either motile or non-motile, Gram-positive rod-shaped lactic acid bacteria that produce mostly L-lactic acid by fermentation from glucose [3]. At present the genus contains 11 species with validly published names, which can be roughly divided into two groups. As the genus name implies, most *Carnobacterium* species (*Carnobacterium divergens**,**Carnobacterium gallinarum**,**Carnobacterium inhibens**,**Carnobacterium jeotgali**,**Carnobacterium maltaromaticum**,**Carnobacterium mobile**,**Carnobacterium viridans*) belong to a group that were originally isolated from biological sources such as living fish or foods derived from animal sources [4]. A second group of *Carnobacterium* spp. has been isolated from cold, low-nutrient environments such as Antarctic ice lakes (*C. funditum**,**C. alterfunditum**,**C. iners*) [5, 6] or Arctic permafrost (*C. pleistocenium**,**C. inhibens* subsp. *gilichinskyi*) [7, 8]. Owing to an upsurge in investigations involving *Carnobacterium* strains isolated from novel environments, at present genome sequences have been published for the following *Carnobacterium* environmental strains: *Carnobacterium* sp. 17–4 isolated from permanently cold sea water [9]; *C. maltaromaticum* strain ATCC 35586 isolated from a diseased salmon [10]; *C. maltaromaticum* strain LMA 28 isolated from ripened soft cheese [11]; and *C. inhibens* subsp. *gilichinskyi* isolated from Siberian permafrost [8, 12]. However, to date only one published report of a genome sequence from a type strain of *Carnobacterium* has appeared, from *C. jeotgali* strain MS3^T^ isolated from salt-fermented shrimp [13]. As part of a larger project to determine the genome sequences of all type strains of the genus *Carnobacterium*, the present study determined the classification and features of *Carnobacterium inhibens* subsp. *inhibens* strain K1^T^ [8] as well as its genome sequence and gene annotations.

## Organism Information

### Classification and features

*Carnobacterium inhibens* subsp. *inhibens* strain K1^T^ ( = DSM 13024^T^ = JCM 16168^T^) is the type strain of the species *C. inhibens* [8, 14]. The strain was isolated from the intestine of an Atlantic salmon [14]. The species epithet was derived from the Latin verb *inhibeo*, meaning “to inhibit”, referring to the growth-inhibitory activity that the bacterium shows [14]. Recent discovery of *C. inhibens* strain WN1359 from Siberian permafrost [15] prompted a re-examination of strains K1^T^ and WN1359, resulting in the proposal to rename the K1^T^ type strain as *C. inhibens* subsp. *inhibens* and the permafrost isolate *C. inhibens* subsp. *gilichinskyi* [8].

**Fig. 1:**
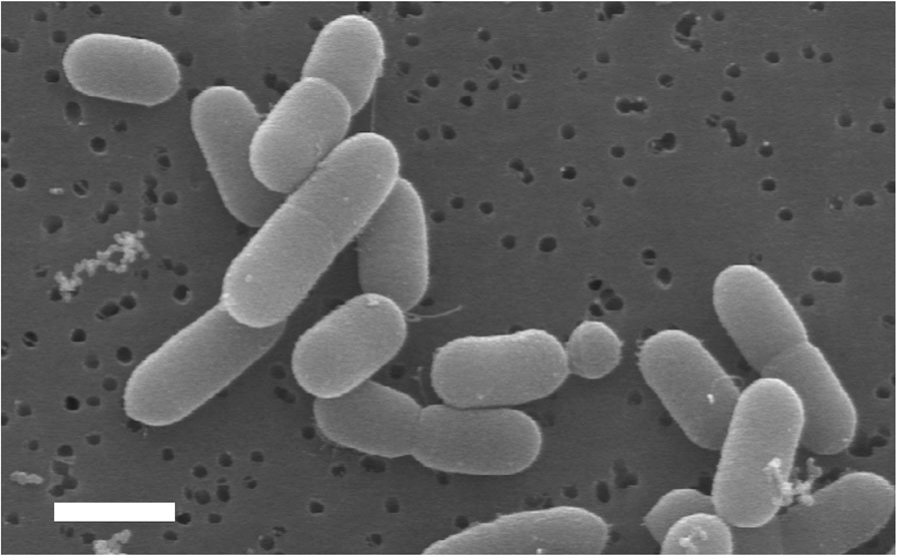
Scanning electron micrograph of *Carnobacterium inhibens* subsp. *inhibens* strain K1^T^. Size bar is 1 μm in length

*Carnobacterium inhibens* subsp. *inhibens* strain K1^T^ is a motile Gram-positive rod (Fig. 1). It is a psychrophile that lacks both catalase and oxidase, does not grow on acetate containing media, but grows at pH 9 and in Trypticase Soy Broth containing up to 6 % (w/v) sodium chloride. Strain K1^T^ is facultatively anaerobic and tryptone as a sole source of nutrient promotes growth. The most abundant cellular fatty acid of strain K1^T^ is oleic acid (18:1*cis9*) [14]. Classification of strain K1^T^ according to the MIGS recommendations published by the Genome Standards Consortium﻿ is presented in Table 1.

**Table 1:** Classification and general features of *Carnobacterium inhibens* strain K1^T^ according to the MIGS recommendations published by the Genome Standards Consortium [20]

MIGS ID	Property	Term	Evidence code^a^
	Current classification	Domain: Bacteria	
		Phylum: *Firmicutes*	TAS [34]
		Class: *Bacilli*	TAS [35, 36]
		Order: *Lactobacillales*	TAS [35, 37]
		Family: *Carnobacteriaceae*	TAS [35, 38]
		Genus: *Carnobacterium*	TAS [1]
		Species: *Carnobacterium inhibens*	TAS [14]
		Subspecies: *Carnobacterium inhibens* subsp. *inhibens*	TAS [8]
		Type strain: K1^T^ (DSM 13024)	
	Gram stain	Positive	TAS [14]
	Cell shape	Rod	TAS [8, 14]
	Motility	Motile	TAS [14]
	Sporulation	Non-spore forming	TAS [8, 14]
	Temperature range	0–37 °C	TAS [8]
	Optimum temperature	35 °C	TAS [8]
	pH range; Optimum	6–9; 8.2	TAS [8]
	Carbon source	Tryptone,	TAS [14]
MIGS-6	Habitat	Gastrointestinal tract of fish (Atlantic salmon)	TAS [14]
MIGS-6.3	Salinity	Grows at 0–6 % NaCl (w/v)	TAS [8, 14]
MIGS-22	Oxygen requirement	Facultative anaerobe; grows better in absence of O_2_	TAS [8, 14, 15]
MIGS-15	Biotic relationship	Unknown	
MIGS-14	Pathogenicity	Unknown	
MIGS-4	Geographic location	Göteborg, Sweden	
MIGS-5	Sample collection	Unknown	
MIGS-4.1	Latitude	Unknown	
MIGS-4.2	Longitude	Unknown	
MIGS-4.3	Depth	Unknown	
MIGS-4.4	Altitude	Below ocean surface	TAS [14]

^a^Evidence codes - *IDA* Inferred from Direct Assay, *TAS* Traceable Author Statement (i.e., a direct report exists in the literature), *NAS* Non-traceable Author Statement (i.e., not directly observed for the living, isolated sample, but based on a generally accepted property for the species, or anecdotal evidence). These evidence codes are from the Gene Ontology project [39]

**Fig. 2:**
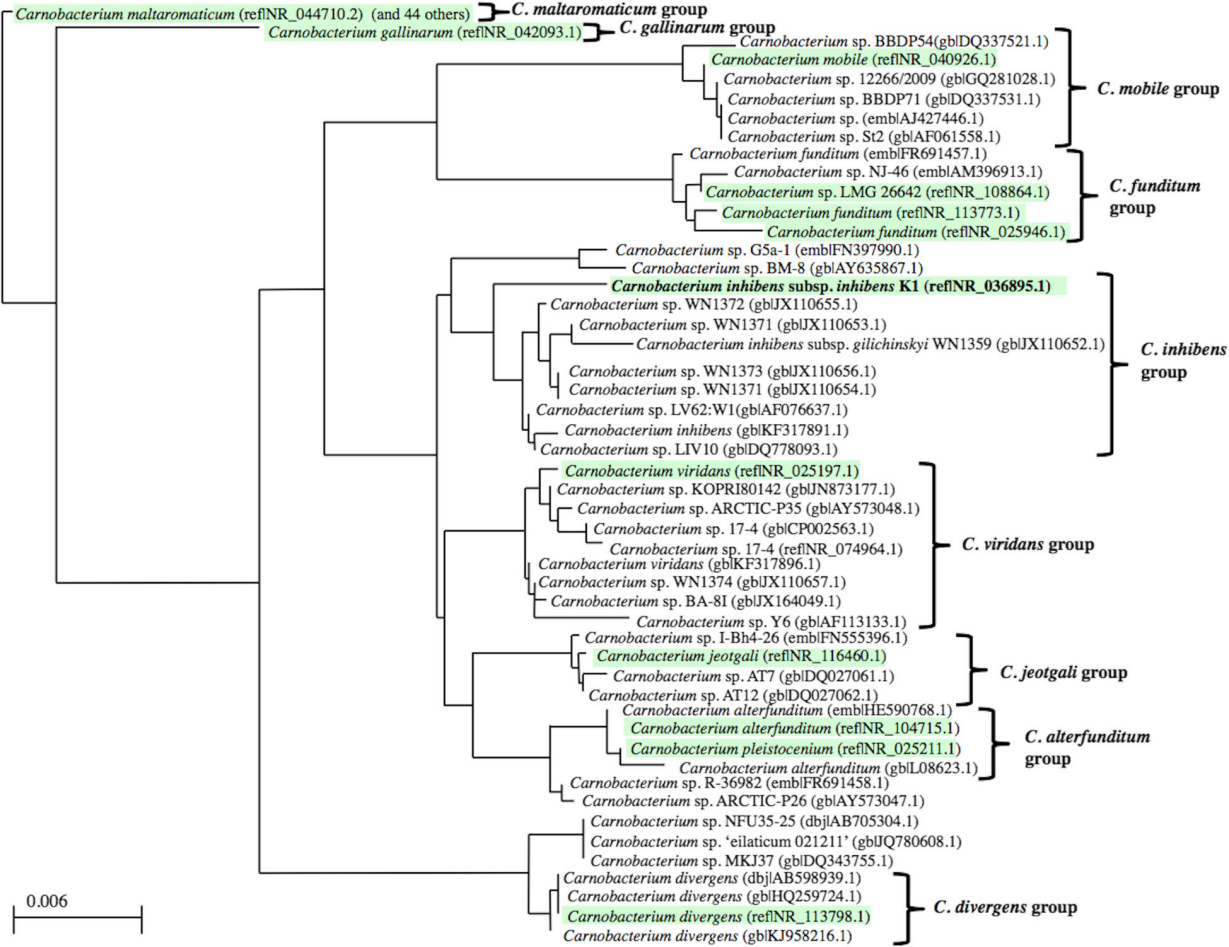
Phylogenetic tree highlighting the position of *Carnobacterium inhibens* subsp. *inhibens* strain K1^T^ relative to other type (*green boxes*) and non-type strains within the genus *Carnobacterium*. Accession numbers are in parentheses: dbj, Database of Japan; emb, EMBL database; gb, NCBI Genbank. The top 100 hits by NCBI-BLASTN were input into the Distance Tree function at NCBI [33]. Alignments were converted to a distance matrix using the Jukes-Cantor distance correction model and the tree was constructed using the Neighbor-Joining method

*C. inhibens* subsp. *inhibens* strain K1^T^ [8] was obtained from the German Collection of Microorganisms and Cell Cultures as strain DSM 13024. The strain was sub-cultured once and was stored as a −70 °C frozen glycerol stock in the corresponding author’s strain collection as strain WN1362. DNA isolated from strain WN1362 corresponding to 16S rRNA gene sequences was PCR amplified with universal bacterial primers B27F (5’-GAGTTTGATCMTGGCTCAG-3’) and B1512R (5’-AAGGAGGTGATCCANCCRCA-3’) as described previously [16] and sequenced at the University of Florida Interdisciplinary Center for Biotechnology Research (UF-ICBR). The sequence was compared with those obtained using NCBI BLAST [17] with the default settings (only highly similar sequences). The most frequently occurring genera were *Carnobacterium* (17 %) and unidentified bacteria (83 %) (100 hits in total). The species with the Max score was *Carnobacterium inhibens* subsp. *inhibens* strain K1^T^ (NCBI Reference Sequence NR_036895) with a shared identity of 100.0 %, thus verifying the identity of strain WN1362 with the type strain. An updated 16S rRNA phylogenetic analysis of *Carnobacterium* spp. isolates including *C. inhibens* subsp. *inhibens* strain K1^T^ is presented in Fig. 2 to supplement and expand upon those published previously [8, 14, 15].

## Genome sequencing information

### Genome project history

This organism was selected for sequencing on the basis of its relevance to environmental issues in phylogenetic diversity, bioenergy, and bioremediation, and is part of the Community Sequencing Program at the U.S. Department of Energy, Joint Genome Institute for projects of relevance to agency missions (http://www.jgi.doe.gov). The project is registered in the Genomes OnLine Database [18] and the permanent draft genome sequence is deposited in GenBank. Draft sequencing and assembly were performed at JGI using state of the art sequencing technology [19]. A summary of the project information is shown in Table 2, which presents the project information and its association with MIGS version 2.0 compliance [20].

**Table 2:** *Carnobacterium inhibens* subsp. *inhibens* strain K1^T^ genome sequencing project details

MIGS ID	Property	Term
MIGS-31	Finishing quality	Improved High-Quality Draft
MIGS-28	Libraries used	PacBio
MIGS-29	Sequencing platforms	PacBio
MIGS-31.2	Fold coverage	273.1×
MIGS-30	Assemblers	HGAP v.2.1.1
MIGS-32	Gene calling method	Prodigal 2.5
	Locus Tag	BR65
	Genbank ID	JQIV01000006.1
	Genbank Date of Release	16 August 2015
	GOLD ID	Gp0042580
	BIOPROJECT	PRJNA234895
MIGS-13	Source material identifier	DSM 13024^T^
	Project relevance	Environmental

### Growth conditions and genomic DNA preparation

Strain K1^T^ was grown to stationary phase by incubation for 36 h at 20 °C in TSY medium without shaking [8]. DNA was isolated from 100 mL of culture using a CTAB bacterial genomic DNA isolation method following the protocol recommended by JGI [21]. DNA fragment size and quality was confirmed by agarose gel electrophoresis and DNA was quantified by fluorometry (Qubit fluorometer, Invitrogen).

### Genome sequencing and assembly

The draft genome of *Carnobacterium inhibens* K1 was generated at the DOE Joint genome Institute using the Pacific Biosciences sequencing technology [19]. A PacBio SMRTbell™ library was constructed and sequenced on the PacBio RS platform, which generated 252,358 filtered sub-reads totaling 752.5 Mbp. All general aspects of library construction and sequencing performed at the JGI can be found at (http://www.jgi.doe.gov). The raw reads were assembled using HGAP (version: 2.1.1) [22]. The final draft assembly contained six contigs in six scaffolds, totaling 2.7 Mbp in size. The input read coverage was 273.1 ×.

### Genome annotation

The assembled sequence was annotated using the JGI prokaryotic annotation pipeline [23] and was further reviewed using the Integrated Microbial Genomes – Expert Review platform [24]. Genes were identified using Prodigal [25], followed by a round of manual curation using GenePRIMP [26] for finished genomes and Draft genomes in fewer than 10 scaffolds. The predicted CDSs were translated and used to search the National Center for Biotechnology Information nonredundant database, UniProt, TIGRFam, Pfam, KEGG, COG, and InterPro databases. The tRNAScanSE tool [27] was used to find tRNA genes, whereas ribosomal RNA genes were found by searches against models of the ribosomal RNA genes built from SILVA [28]. Other non–coding RNAs such as the RNA components of the protein secretion complex and the RNase P were identified by searching the genome for the corresponding Rfam profiles using INFERNAL [29]. Additional gene prediction analysis and manual functional annotation was performed within the Integrated Microbial Genomes platform [23] developed by the Joint Genome Institute, Walnut Creek, CA, USA.

### Genome properties

**Fig. 3:**
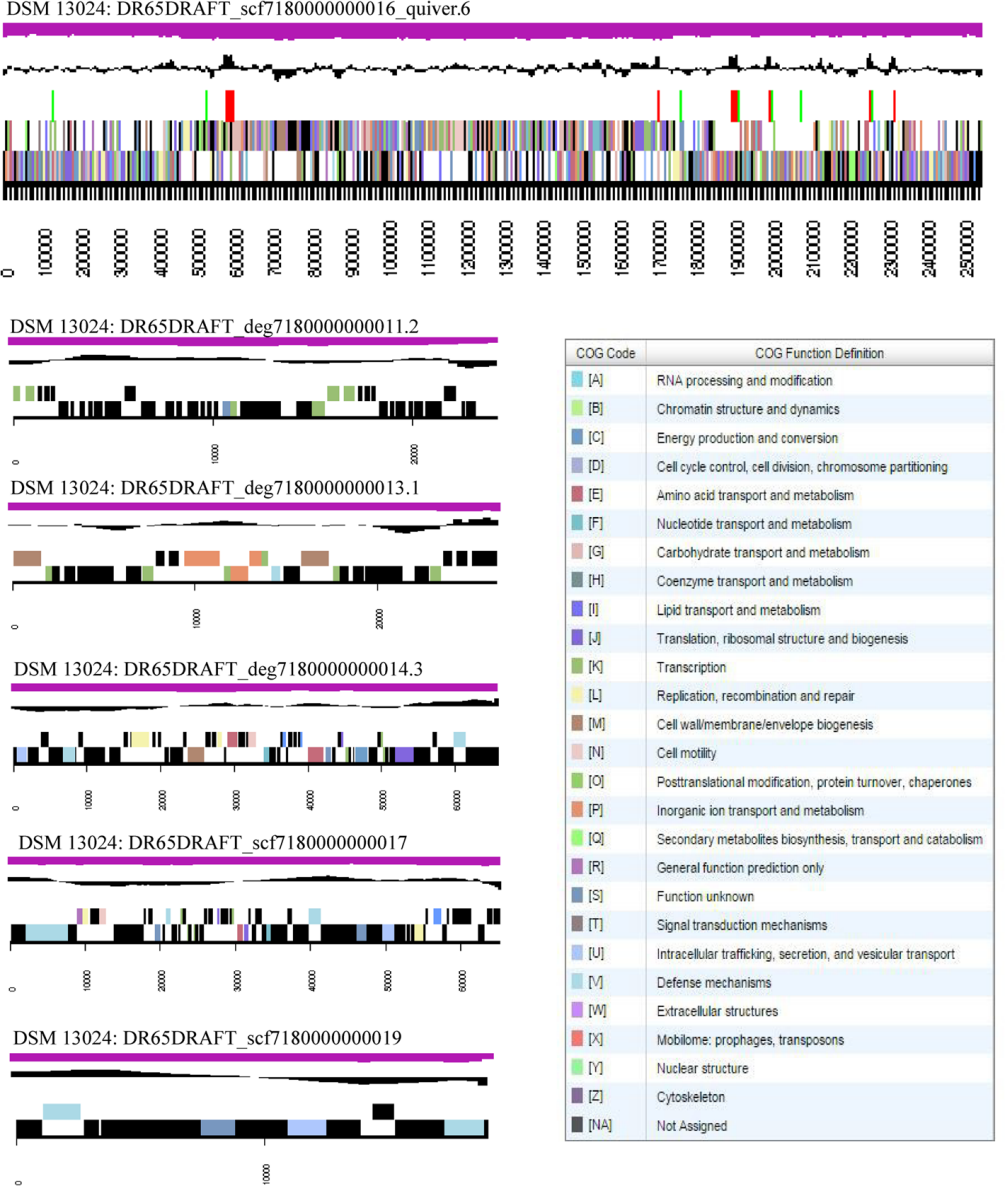
Graphical map of the six scaffolds assembled for the genome of *Carnobacterium inhibens* K1T, DSM 13024. From top to bottom, the scaffolds are: DSM 13024: DR65DRAFT_scf7180000000016_quiver.6, DSM 13024: DR65DRAFT_deg7180000000011.2, DSM 13024: DR65DRAFT_deg7180000000013.1, DSM 13024: DR65DRAFT_deg7180000000014.3, DSM 13024: DR65DRAFT_scf7180000000017, and DSM 13024: DR65DRAFT_scf7180000000019. From bottom to the top of each scaffold: Genes on forward strand (color by COG categories), Genes on reverse strand (color by COG categories), RNA genes (tRNAs *green*, rRNAs *red*, other RNAs *black*), GC content, GC skew

The genome includes five smaller contigs, for a total size of 201,043 bp, and one large contig of 2,547,565 bp (34.85 % GC content) (Fig. 3). For the genome, 2737 genes were predicted, 2621 of which are protein-coding genes. Of these, 2151 were assigned to a putative function with the remaining 470 genes annotated as hypothetical proteins. 1838 protein coding genes belong to paralogous families in this genome, corresponding to a gene content redundancy of 67.15 %. The statistics of the genome are summarized in Tables 3 and 4. Examination of the sequence data for the five small contigs revealed a variety of putative genes encoding plasmid functions such as: autonomous replication, mobilization, bacteriocin production and immunity, toxin-antitoxin systems, and Hg or Cd/Co resistance cassettes; therefore it is reasonable to assume that these five small contigs represent plasmids.

**Table 3:** Genome statistics

Attribute	Value	% of Total
Genome size (bp)	2,748,608	100.00
DNA coding (bp)	2,356,497	85.73
DNA G + C (bp)	957,950	34.85
DNA scaffolds	6	100.00
Total genes	2737	100.00
Protein coding genes	2621	95.76
RNA genes	116	4.24
Pseudo genes	66	2.41
Genes in internal clusters	515	18.82
Genes with function prediction	2151	78.59
Genes assigned to COGs	1900	69.42
Genes with Pfam domains	2196	80.23
Genes with signal peptides	113	4.13
Genes with transmembrane helices	691	25.25
CRISPR repeats	0	0

**Table 4:** Number of genes associated with general COG functional categories

Code	Value	% age	Description
J	196	9.36	Translation, ribosomal structure and biogenesis
A	25	1.20	RNA processing and modification
K	186	8.89	Transcription
L	101	4.83	Replication, recombination and repair
B	19	0.91	Chromatin structure and dynamics
D	32	1.53	Cell cycle control, Cell division, chromosome partitioning
V	71	3.39	Defense mechanisms
T	78	3.73	Signal transduction mechanisms
M	113	5.40	Cell wall/membrane biogenesis
N	51	2.44	Cell motility
U	22	1.05	Intracellular trafficking and secretion
O	61	2.91	Posttranslational modification, protein turnover, chaperones
C	71	3.39	Energy production and conversion
G	186	8.89	Carbohydrate transport and metabolism
E	163	7.79	Amino acid transport and metabolism
F	96	4.59	Nucleotide transport and metabolism
H	76	3.63	Coenzyme transport and metabolism
I	80	3.82	Lipid transport and metabolism
P	102	4.87	Inorganic ion transport and metabolism
Q	34	1.62	Secondary metabolites biosynthesis, transport and catabolism
R	199	9.51	General function prediction only
S	156	7.45	Function unknown
-	837	30.58	Not in COGs

## Conclusion

*Carnobacterium inhibens* is widely distributed in the environment, having been isolated from Atlantic salmon [14, 30], biogas slurry [31], a medicinal plant [32], and Siberian permafrost [8, 15]. In this communication we report an improved high-quality draft genome sequence of *Carnobacterium inhibens* subsp. *inhibens* strain K1^T^ ( = DSM 13024^T^ = JCM 16168^T^). Genome analysis of this strain demonstrated a single presumed chromosome and at least five putative extrachromosomal elements.
